# Neoatherosclerosis causing edge in-stent restenosis: optical coherence tomography findings

**DOI:** 10.1007/s12471-015-0680-y

**Published:** 2015-04-08

**Authors:** F. Alfonso, J. Restrepo, J. Cuesta, T. Bastante, F. Rivero, A. Benedicto

**Affiliations:** Cardiac Department, Hospital Universitario de La Princesa, Instituto de Investigación Sanitaria, IIS-IP, Universidad Autónoma de Madrid, c/ Diego de León 62, 28006 Madrid, Spain

**Keywords:** Edge in-stent restenosis, Optical coherence tomography, Neoatherosclerosis

## Abstract

A patient presenting with ‘edge’ in-stent restenosis 12 years after the implantation of a bare-metal stent in the mid-left anterior descending coronary artery is described. Optical coherence tomography disclosed the presence of ruptured neoatherosclerosis at the stent edge. The value of this imaging technique to unravel this unique underlying anatomic substrate is discussed. The therapy of choice for patients presenting with edge in-stent restenosis (ISR) is reviewed.

Treatment of patients presenting with in-stent restenosis (ISR) remains a challenge [1]. Neoatherosclerosis may constitute the underlying substrate of ISR [2]. We present a patient who developed very late ‘edge ISR’ caused by neoatherosclerosis.

A 63-year-old man presented with effort angina. Twelve years before, he received a bare-metal stent (BMS) in the left anterior descending coronary artery. Ten years later, repeated angiography showed an excellent stent result. Currently, coronary angiography showed a tight lesion at the distal edge of the stent (Fig. 1a). Optical coherence tomography (OCT) revealed mild, uniform, neointimal tissue proliferation along the stent. However, its distal segment showed neoatherosclerosis which, near the stent edge, progressed into a ruptured occlusive fibroatheroma with thrombus (Fig. 1b–d). Immediately distal to the stent edge, a large lipid plaque was also recognized (Fig. 1e). A drug-eluting stent (DES) was successfully implanted. Repeated OCT confirmed excellent stent expansion and apposition, but unravelled multiple areas with plaque prolapse and residual thrombus (Fig. 2).

**Fig. 1:**
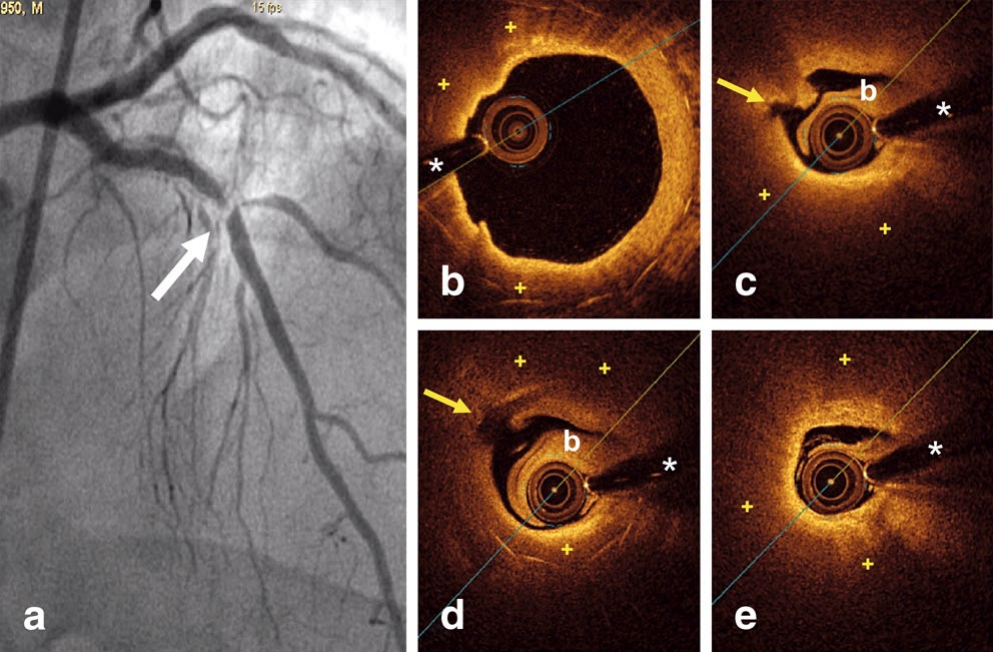
**a** Coronary angiography revealing a severe in-stent restenosis at the distal edge of the stent (*arrow*). **b–e** OCT before intervention. **b** Distal segment of the stent showing a glistening neointima covering dark tissue (+) overlying the stent struts. **c–d** Ruptured fibroatheroma (*yellow arrow*) with some protruding thrombus nearly completely obscuring the stent struts (only visualized in **d** from 4 to 7 o’clock), (*b* residual blood). **e** Occlusive lipid plaque (+) immediately distal to the stent edge. (+)= Lipid pools. (*)= indicates wire artifact

**Fig. 2:**
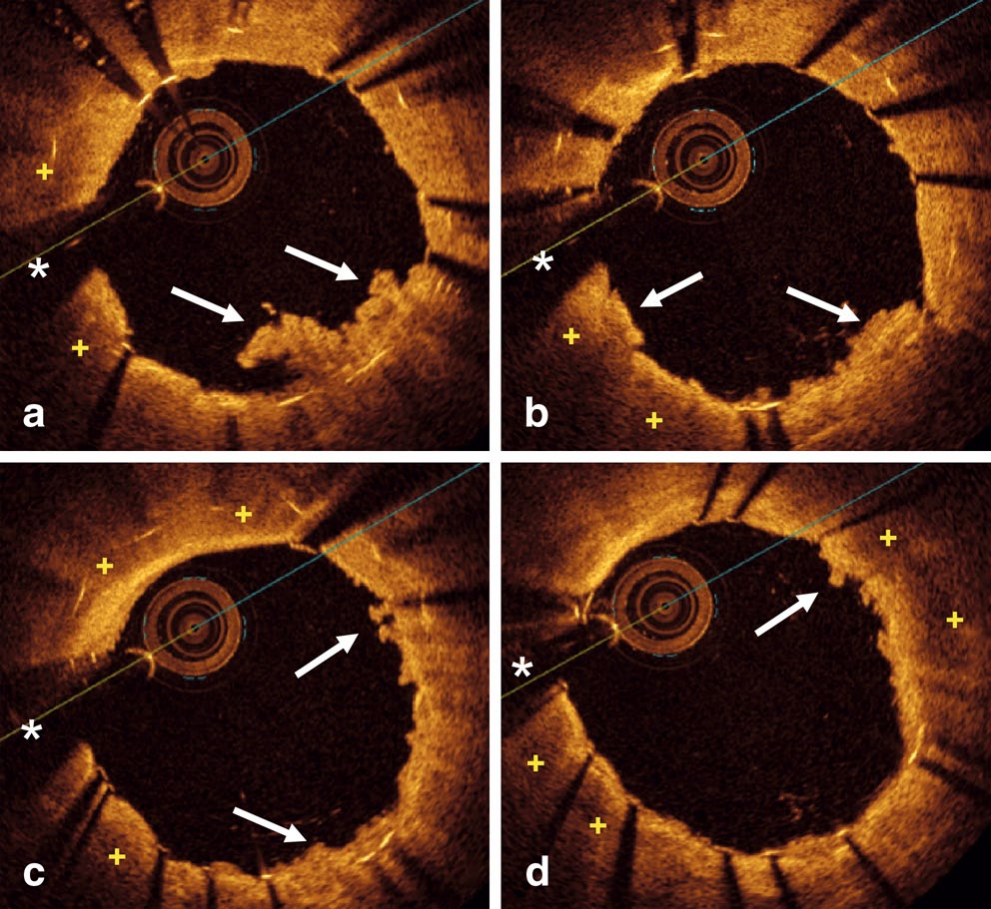
OCT findings after DES implantation. A nicely expanded stent with multiple areas of tissue prolapse (*white arrows*) is depicted (**a–c**). A double stent layer can be visualized with residual lipid tissue (+). **d** New stent, extending beyond the previous stent, disclosing the underlying lipid plaque (+) and prolapsing tissue (*white arrow*). (*) = indicates wire artifact

Neoatherosclerosis occurs less frequently and later in patients receiving BMS as compared with those treated with DES [1, 2]. Complicated neoatherosclerosis (rupture of a thin-cap fibroatheroma) may explain unstable clinical presentations in patients with ISR and in those with very-late stent thrombosis [2]. Due to its spatial resolution (15 μm), OCT represents the technique of choice for the diagnosis of neoatherosclerosis [3]. Edge-ISR occurs more frequently in patients with DES-ISR than in those with BMS-ISR [4] and repeat stenting has been advocated in this setting [5]. However, to the best of our knowledge, complicated neoatherosclerosis causing edge-ISR has not been previously reported.

## Funding

None.

## Conflict of interest

None declared.
